# Microstructural Evolution during Accelerated Tensile Creep Test of ZK60/SiC_p_ Composite after KoBo Extrusion

**DOI:** 10.3390/ma15186428

**Published:** 2022-09-16

**Authors:** Yang-Yang Wang, Chen Jia, Morteza Tayebi, Bejan Hamawandi

**Affiliations:** 1 School of Engineering, Xi’an Siyuan University, Xi’an 710038, China; 2 Xi’an Aerospace Propulsion Test Technology Institute, Xi’an 710100, China; 3 Young Researchers and Elites Club, Science and Research Branch, Islamic Azad University, Tehran 14778-93855, Iran; 4 Department of Applied Physics, KTH Royal Institute of Technology, SE-106 91 Stockholm, Sweden

**Keywords:** ZK60/SiCp composite, dynamic precipitation, dynamic recrystallization, double twinning, double Friedel–Escaig mechanism, accelerated creep test

## Abstract

In the current study, the creep properties of magnesium alloy reinforced with SiC particles were investigated. For this purpose, ZK60/SiCp composite was produced by the stir casting method following the KoBo extrusion and precipitation hardening processes. The creep tests were performed at 150 °C under 10–110 MPa. The results showed that the stress exponent (n) and the average true activation energy (Q) was changed at high stresses, was found with increasing stress, the creep mechanism changing from grain boundary sliding to dislocation climb. The results of microstructure characterization after the creep test showed that at low stresses, the dynamic recrystallization resulting from twinning induced the GBS mechanism. However, at high stresses, with increasing diffusion rates, conditions are provided for dynamic precipitation and the dislocation climb of the dominant creep mechanism. Examination of the fracture surfaces and the surrounding areas showed that the cavity nucleation in the ternary boundary and surrounding precipitation was the main cause of damage. The evaluation of the samples texture after creep showed that the unreinforced alloy showed a moderately strong fiber texture along the angle of ϕ_1_ = 0–90°, which was tilted about Φ = 10°. A new strong texture component was observed at (90°, 5°, 0°) for the composite sample, which crept due to minor splitting of the basal pole by ~5° toward RD.

## 1. Introduction

Concerns about increased greenhouse gases and increased fuel consumption are pushing us towards advanced lightweight materials [1,2,3]. In the automotive industry, lightweight parts are required, which can endure operating temperatures around 150 °C. Low density Mg alloys are a serious choice for these materials. However, magnesium alloys have low strength due to their large grains due to the casting process. For this purpose, it is necessary to employ methods to increase the strength and reach a proper strength to weight [4,5]. Compositing can be a good option for strengthening. The composite controls the mechanical properties of high temperatures through two methods of direct and indirect strengthening. Reinforcements are a critical role in direct strengthening, which is achieved through effective load transfer. Studies [6] have shown that by enhancing the reinforcement length-to-diameter ratio, the load transfer increases, making the fiber and whisker reinforcement more effective than the particle. However, the high price of whiskers and special methods for uniform distribution has limited its applications. Indirect strengthening results from changes in the matrix microstructure of the composite by the addition of reinforcement. Indirect reinforcement can be attributed to the threshold stress resulting from the presence of reinforcement and precipitation in the microstructure leading to the interaction between the dislocation and the second phase particles [6,7]. Indirect strengthening has a direct relationship with the matrix alloy. Initially, Mg-Al alloys were considered due to their high castability and the presence of binary and ternary intermetallic compounds. This alloy system has a strong aging effect and there are thermally stable intermetallic compounds in the microstructure, but due to the segregation of soluble elements into grain boundary and dendrites, eutectic phases are formed which cause the reduction in the melting temperature of these regions to compare to the intra-grain areas [8,9,10]. It decreases and reduces the strength of dendrites and grain boundaries, affecting the creep properties of magnesium alloys. It is not easy to prevent soluble elements from segregating into grain boundary and dendrites during solidification. The weakening of boundaries is due to the segregation of soluble elements, a serious factor in the creep properties of magnesium alloys [11]. For this purpose, the focus of the studies was on the Mg-Zn alloy system with no low temperature phases occurring in its grain boundaries. In addition, the addition of Zn to magnesium results in a fine grain structure and improves the corrosion properties and strength of the alloy. The presence of Zn in the Mg microstructure also stabilizes and widens the dislocations, which reduces cross-slip and climb, and subsequently reduces creep rate [12,13]. Adding Zn to Mg decreases the grain size, but this refinement is not sufficient to achieve acceptable strength according to the Hall–Petch relation [14]. For this purpose, the Zr element was added to the alloy for acceptable refining, known as ZK60 [15,16,17]. Zr acts as a nucleation agent during casting due to its high melting temperature compared to Mg and Zn and causes the alloy refinement. On the other hand, due to its larger atomic radius than Mg and Zn, its diffusion rate is low in the Mg alloy and causes solution hardening [18,19,20,21]. In addition to the alloying method, the extrusion process can be used to achieve fine structure and high strength. The strength created by alloying and extrusion responds to mechanical properties at room temperature, but the major applications of light alloys at high temperatures are to reduce the stability of the structure by increasing the operating temperature. To create a stable high temperature structure, alloys of excess solubility are used to control structural changes by creating precipitation. In the Mg-Zn-Zr alloy system, excessive solubility of Zn and Zr in Mg causes (Mg, Zn) and (Zn, Zr) precipitates in the microstructure, which, on the one hand, inhibits grain growth at high temperatures and on the other hand, interacting dislocations with the precipitation improve the high temperature strength of the alloy [19,22,23,24]. Various heat treatment methods, such as T6, T5, and T4, have been reported to achieve a relatively homogeneous distribution and an effective morphology of ZK60 alloy precipitates.

As mentioned, in recent years, magnesium alloys have been studied for high-temperature applications, and creep-resistant alloys are industrialized and employed for commercially application. However, compared to other alloys, the demand for Mg alloys with high creep resistance is quite low. This is related to unfavorable performance compared to steels and Al alloys and high costs, especially Mg alloys containing RE, such as WE54. Therefore, the designation of modern creep-resistant magnesium casting alloys with higher efficiency and low production cost will be developed in near future. The applications of Mg alloys with high creep-resistance include automotive, aerospace, and defense industries, where the use of lightweight materials is vital. Due to the exclusive benefits of these materials, alloys such as, AM60 and AZ91 can offer similar performance to A380 aluminum alloy for structural applications at low operating temperatures, such as automotive bodies and chassis with significant lower weights. Yet, components used in powertrains, i.e., transfer cases and engine cases with operating temperatures close to 150 °C, require special high-creep-resistant magnesium alloys [25,26]. In addition, these types of alloys have the potential to be used in aerospace industries that require weight reduction in parts that still maintain high creep resistance at high temperatures. According to the literature, it is clear that the creep mechanisms in magnesium alloys are different according to alloy systems and the test conditions. Under low stress at relatively low temperatures, Mg-Al alloys creeping at 100–175 °C, grain boundary diffusion (GBD) and grain boundary sliding (GBS) mechanisms are dominant. At higher stress in AM-SC1 alloys, GBS is also the main creep mechanism [27]. Under low stresses and at higher than 300 °C, GBD is also the dominant creep mechanism of Mg-Sc-Mn alloys tested [28]. Dislocation climb typically dominates creep in magnesium alloys under moderate to high stresses and moderate temperatures. Non-basal slip, such as cross slip, typically causes alloys to creep at relatively high temperatures and under low stresses. At stresses depending on the yield strength of the alloy, a cooccurrence of dislocation glide and climb causes creep deformation. For example, alloy AS21 creeps at 150 °C under 70 to 100 MPa, dislocation climb and glide dominate because the stress is greater than the yield strength of the alloy [29]. Based on the author’s knowledge, no comprehensive data on the high temperature creep behavior of the KoBo extruded and aged ZK60 alloy and ZK60/SiC_p_ composite have been reported so far. The purpose of this study was to investigate the microstructure evaluation during the creep test of ZK60 alloy and ZK60/SiC_p_ composite via grain refinement and stabilization of microstructure by KoBo extrusion and the aging process.

## 2. Materials and Methods

### 2.1. Materials

Magnesium alloy containing 5.1% Zn and 0.48% Zr as composite matrix and of 10% volume fraction SiC particles with a diameter less than 40 μm with a purity of 99%, % was used as the reinforcement phase.

### 2.2. Composite Preparation

The composite was produced by the stir casting method. The alloy ingot was first melted in the induction furnace at 760 °C under CO_2_ + SF_6_ atmosphere. After homogenization of the alloy, SiC particles were added to the melt by vortex method and stirred for 15 min with a titanium stirrer at 320 rpm. Finally, the composite was cast into a steel mold. It should be noted that the mold was preheated to 150 °C before casting. To compare the results, ZK60 alloy was also cast under similar conditions to the composite. The details of casting method are described in [1].

### 2.3. Heat Treatment and KoBo Extrusion

**Figure 1:**
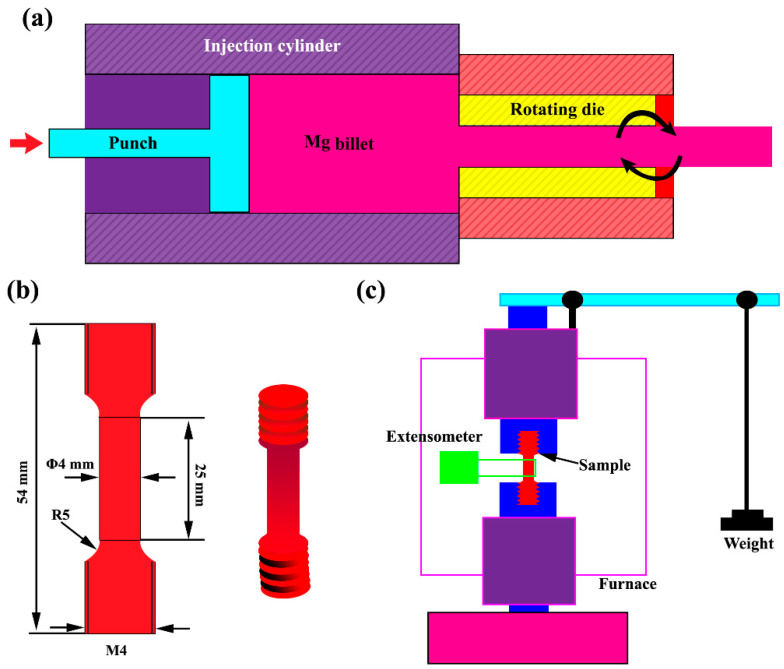
Schematic of the: (**a**) KoBo extrusion method, (**b**) creep sample, and (**c**) creep setup.

The resulting sample was homogenized at 400 °C for 8 h and then quenched in water. The samples were extruded with forward–backward rotating die which called KoBo method at room temperature. The details of the KoBo method is described in [30]. Furthermore, the KoBo extrusion schematic is brought in Figure 1a. The extrusion was performed under 0.1 mm/s punch speed with 5 Hz die oscillation frequency at 7° die rotation angle. Furthermore, precipitation hardening treatment was carried out at 175 °C for 12 h as pre aging. The details of the optimum time and temperature of precipitation hardening are described in [23].

### 2.4. Creep Test

The creep test was performed by the accelerated creep test method at 150 °C and at stress levels (10, 20, 30, 40, 50, 60, 70, 80, 90, 100, and 110 MPa) below the yield stress [31,32]. The mechanical properties of the samples are brought in Table 1. To calculate the minimum creep rate under various stress levels, a step-by-step load increment was performed on a specimen. In order to determine the value of the activation energy, creep tests were performed at 30 and 60 MPa stresses at three temperatures of 150, 200, and 250 °C. The creep test was performed with the SANTAM model STM-100 according to ASTM EB9-83 standard. The dimensions of the specimens were selected according to ASTM E8 standard with 4 mm φ × 20 mm gauge length (Figure 1b). A back-strain model with linear extensometer sensor with an accuracy of 0.001 mm was used to calculate the sample length elongation during the test. The schematic of the creep setup is brought in Figure 1c.

### 2.5. Characterizations

The X-ray diffraction (XRD) analysis was carried out using a Philips XRD machine (model PW1800) at a voltage of 30 kV with CuKα spectrum and 1.54056 Å wavelength. The microstructure of the specimens after conventional metallographic operation was examined by Philips optical microscope (OM) and scanning electron microscope (SEM, Philips model XI30), which was equipped with EDS elemental analysis. For determination of grain size, etch solution [33], 5 mL acetic acid, 6 g picric acid, 10 mL water, and 100 mL 95% ethanol were used.

## 3. Results and Discussion

### 3.1. Microstructure before Creep Test

**Figure 2:**
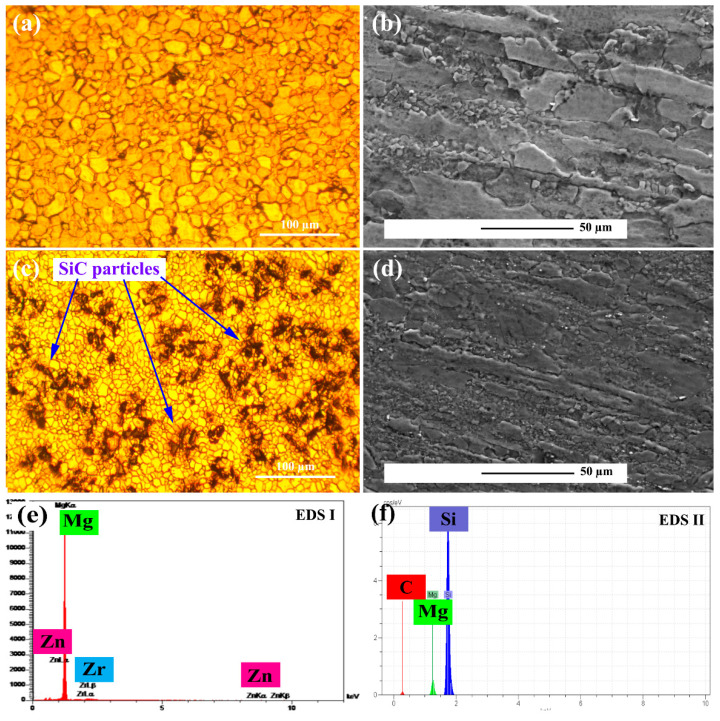
Microscopic image after extrusion: (**a**) unreinforced alloy perpendicular to the extrusion direction, (**b**) unreinforced alloy in the directional of extrusion, and (**c**) ZK60/SiC_p_ composite perpendicular to the extrusion direction, (**d**) ZK60/SiC_p_ composite in the directional of extrusion, (**e**) EDS analysis of the matrix of the ZK60/SiC_p_ composite, and (**f**) EDS analysis of the matrix of the ZK60/SiC_p_ composite.

Figure 2a illustrates the OM micrograph of unreinforced alloy perpendicular to the extrusion direction. The figure shows that the unreinforced alloy has coaxial grains with an average grain size of 10 μm. Figure 2b, shows the SEM micrographs of the unreinforced alloy in direction of the extrusion. According to the figure, it is obvious that bimodal grain distributions are achieved during the extrusion process. Some grains are elongated in the extrusion direction and partial recrystallization occurred, and grain size was reduced to 5 μm. Figure 2c shows the OM micrograph perpendicular to the extrusion direction of the ZK60/SiCp composite with an average grain size of 5 μm. Also shown in Figure 2d are elongated grain and recrystallization grains with lower than 1–2 μm grain size. By comparing Figure 2b,d it is clear in the composite microstructure higher deformation degree induced higher grain recrystallization. Figure 2e,f show the EDS analysis of the matrix and reinforcement which indicate during the production process oxidation did not occur.

**Figure 3:**
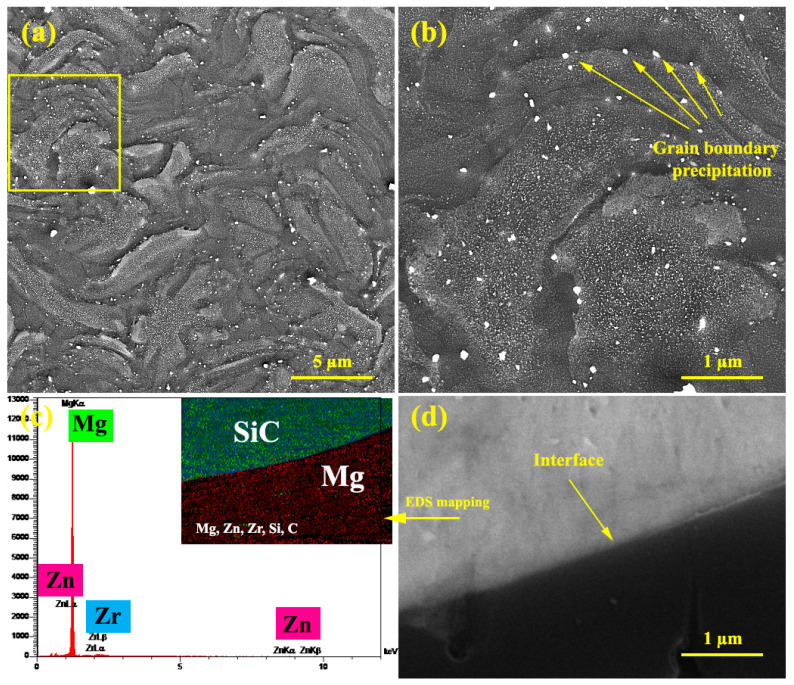
Microscopic image after extrusion and precipitation hardening processes: (**a**,**b**) unreinforced alloy, (**c**) EDS analysis, and (**d**) particle/matrix interface.

Figure 3a,b show the unreinforced alloy microstructure after extrusion and precipitation hardening. According to the figure, twisted-shape grain morphology is observed, which is caused by die rotation during KoBo extrusion. Furthermore, the distribution of white precipitates is also observed in grains. Elemental analysis shows that the precipitates are of types (Mg, Zn) and (Zn, Zr). In the higher magnification figure, it is observed that in the precipitates near the grain boundaries are grown. Also shown in Figure 3d are particles with no cracks, voids, or cavities around them indicating suitable particle bonding with the matrix. A strong interface provides the conditions for effective load transfer [34].

### 3.2. Creep Data

#### 3.2.1. Creep Curve

**Figure 4:**
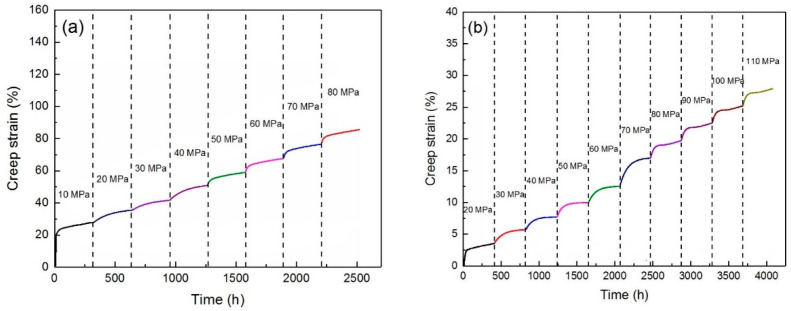
Creep diagram at 150 °C at different stress levels: (**a**) unreinforced alloy, and (**b**) ZK60/SiC_p_ composite.

**Figure 5:**
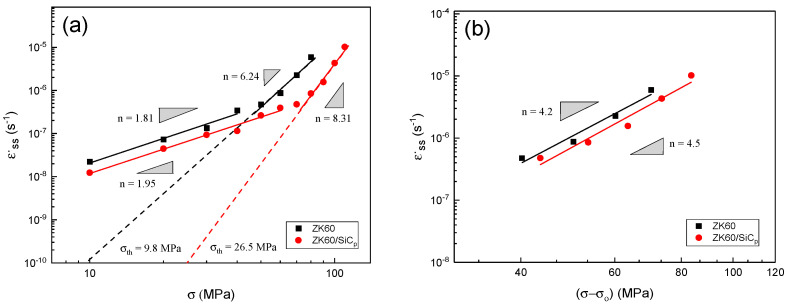

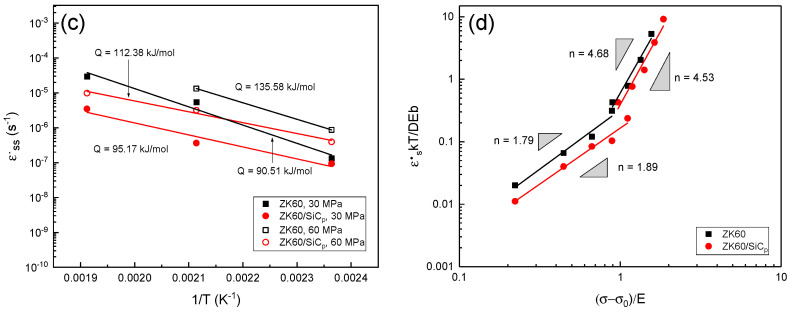
Minimum creep rate changes in unreinforced alloy and ZK60/SiC_p_ composite in terms of: (**a**) stress and (**b**) effective stress, (**c**) Minimum creep rate changes in 1/T at 30 and 60 MPa for unreinforced alloy and ZK60/SiC_p_ composite, and (**d**) Normalized minimum creep rate variations with normalized stress diffusion coefficient for unreinforced alloy and ZK60/SiC_p_ composite.

Figure 4a illustrates the unreinforced alloy creep graph under 10–80 MPa at 150 °C. In the first part of the diagram, the initial creep stage is detectable and very short, after a momentary deformation at initial loading. It is evident that with increasing time, the creep rate has gradually decreased to a constant value, which is the beginning of the second creep stage, or steady-state creep. The characteristic of this step is the linearity of the strain changes over time. In fact, it can be said that a balance has occurred between the mechanism of work hardening and recovery [35]. The conditions at each stress level are similar so that with increasing load the initial stage is observed and then the linear stage is detectable. At the end of the graph, the slope of the curve is increased to a fracture, which represents the third stage of creep. In Figure 4b, the creep behavior of the ZK60/SiC_p_ composite is plotted at 150 °C at different stress levels. It can be seen from the figure that the composite sample behaves like an alloy except that the composite sample can withstand up to 110 MPa stress. By comparing Figure 5a,b, it can be seen that the composite sample was withstand up to 37.5% more load than the unreinforced alloy, which is related to the higher load to transfer.

#### 3.2.2. Minimum Creep Rate

Based on Equation (1) from time-dependent strain variation curve, steady-state creep stage slope was derived, and its results were calculated as a double logarithmic graph in terms of applied stress as shown in Figure 5a. The data were fitted by the best line and the stress exponent was determined based on the slope of the lines.

(1) $$\dot{\varepsilon_{s}}={A\sigma }^{n}\exp(\frac{-Q}{\mathrm{RT}})$$

where A, σ, Q, T, and R are the equation constant, applied stress, activation energy, temperature, and universal gas constant, respectively.

As shown in Figure 5a, it is apparent that under stresses lower than 50 MPa, the stress exponent of the unreinforced alloy is equal to 1.81 and within the GBS mechanism. At higher stresses than 50 MPa, the stress exponent increased abruptly to 6.24, indicating that by increasing the stress, the creep mechanism changed. The stress exponent is approximately 7 related to the dislocation climb [36]. The general trend of composite stress exponent changes is near to that of alloys, so that up to 60 MPa, the stress exponent is 1.95, and with increasing stress from 110 MPa thereafter, the stress exponent is markedly increased to 8.31. Such behavior has also been reported [34] for ZK60 matrix composites reinforced with SiC whiskers, in which the n values at low stress for ZK60 alloy was 2.03 (n = 2.11 for composite) and by increasing the load was increased to 6.49 (n = 12.96 for composite). The higher tensile strength of the composite than that of the unreinforced alloy can be related to the presence of particles in the microstructure. As in composites, in addition to matrix precipitation, particles also perform the role of creep-rate-controlling factor by pinning the grain boundaries via creating a barrier for dislocation movement [37].

#### 3.2.3. Threshold Stress

The increase in n value at high stresses can be due to the interaction of dislocations with the precipitation in the alloy microstructure and the interactions of the dislocations with the precipitation and particles in the composite microstructure. The threshold stress was calculated for unreinforced alloy and composite. To calculate the threshold stress, the fit-line on the minimum creep data was extended to 10^−10^/s. In Figure 5a, the determined threshold stress for the alloy and composite are 9.8 MPa and 26.5 MPa, respectively, indicating that the threshold stress was significantly increased by compositing. By comparing the results with another study [34] the difference between the results of this study and the results reported for the ZK60/SiC_w_ composite is higher threshold stresses, which due to different strengths of reinforcements type (whisker and particle) as well as different grain sizes. It is also illustrated in Figure 5a that the composite sample curves have shifted to higher stresses than the unreinforced alloy, indicating that the presence of reinforcements in the composite not only increased the threshold stress but also improved the creep strength of the material.

To determine the active mechanism during creep, the threshold stress (σ_0_) was lower than the applied stress to obtain the effective stress. By plotting the minimum creep rate in terms of the effective stress in Figure 5b, it is apparent that the n of the unreinforced alloy has decreased from 6.24 to 4.2, indicating the mechanism of dislocation climb, and the n calculated with studies on the AM50 alloy die cast (corresponds to stresses above 100 MPa at temperatures of 150–225 °C) [38]. In fact, the presence of precipitation and its interaction with dislocation is a major factor in increasing the n. It can be seen from Figure 5b that the strength of the composite has decreased from 8.31 to 4.5. By comparing the true n values at high stresses for the unreinforced and composite specimens, their n is closer. In fact, calculating the effective stress eliminates the influence of the presence of precipitation and reinforcement from the calculations to bring the amount of n closer to the matrix alloy.

#### 3.2.4. Activation Energy

Figure 5c depicts the activation energy of alloy and composite samples crept under 30 and 60 MPa at 150–250 °C. As can be seen at 30 MPa stress the apparent activation energy (Q_a_) of the alloy and composite is 90.51 and 95.17 kJ/mol, respectively, and under 60 MPa the Q_a_ for the alloy is 135.58 kJ/mol. For the composite at 60 MPa, the Q_a_ is calculated to be 112.38 kJ/mol, which is less than the lattice self-diffusion of magnesium (135 kJ/mol [36]). The decrease in activation energy can be attributed to the increase in the diffusion rate of the alloy and the occurrence of recrystallization. The activation energy has been reduced by creating a refinement structure due to recrystallization [39]. To eliminate the elastic modulus interdependency on the test temperature, the true activation energy was calculated from Equation (2), assuming that the alloy creep follows a simple power-law creep [40].

(2) $$Q_{c}=Q_{a}+\mathrm{RT}\left[1+\frac{\left(n-1\right)T}{E}\left(\frac{\partial E}{\partial T}\right)\right]$$

where Qc is the true activation energy, and E is the elastic modulus.

The calculated true Q_c_ values for the unreinforced alloy and composite at low stresses were 60.28 and 60.74 kJ/mol, respectively, and for the high stresses were 95.09 and 93.44 kJ/mol, respectively, which is less than the self-diffusion in Mg. The calculated activation energy at low stresses for the unreinforced alloy and composite is within the GBS mechanism [36,41]. Due to the fine-grained unreinforced alloy and composite and the high grain boundary fractions in the microstructure obtained during the KoBo extrusion, GBS can be easily activated in these conditions [36]. Similar results have been obtained for the Q value of AZ91 alloy by Pekguleryuz and Celikin (σ = 50 MPa, T = 125–175 °C, n = 2, and Q = 45–30 kJ/mol) [42]. At high stresses, the Q value for the alloy and composite is in the range of pipe diffusion mechanism and dislocation climb, and given the calculated threshold stress it is likely that the mechanism of the climb is dominant over pipe diffusion. Kunst et al. [43] stated a Q of AJ62 + Sr alloy 92 kJ/mol in collaboration with the dislocation climb mechanism, which was explained by the presence of nanometer precipitates in the microstructure and the dislocation interaction.

#### 3.2.5. Mechanism

The minimum creep rate was normalized by the diffusion coefficient of the alloy at the test temperature according to Dorn’s relation to investigate the resistance of the alloy to creep independent of the test temperature. The normalized steady creep rate ($\dot{\varepsilon }\mathrm{kT}/\mathrm{DEb}$) versus the normalized effective stress ($\sigma -\sigma_{{}^{\circ}}/E$) are plotted in Figure 5d. For calculations b is the magnesium Berger’s vector (b = 0.32 nm [44]), k is the Boltzmann constant (1.38 × 10−23 J/K), D is the diffusion coefficient of magnesium on the basis of the relation $D=D_{0}\times \exp\;\left(-Q/\mathrm{RT}\right)$ where D_0_ equals 10^−4^ m^2^s^−1^ [44], E is the elastic modulus at test temperature and T is the test temperature (423 K). By fitting the line to the obtained data, at low stresses, the n value was measured to be 1.79 for the alloy and 1.89 for the composite, ranging from 1 to 2, which is in good agreement with the Ball–Hutchinson model and the GBS mechanism [45]. Nabarro–Herring and Coble creep fine-grained materials commonly contribute to deformation at or near the grain boundary and are considered as induced GBS mechanisms [36]. Due to the grain size of the unreinforced alloy (lower than 10 µm) and the composite (lower than 5 µm) conditions for GBS at low stresses through a diffusive mechanism is likely. The grain boundary slide is closely related to the boundary energy. In other words, a high-energy grain boundary tends to increase GBS. Due to the extrusion of the specimens before the creep test, the elongated grains are oriented in the extrusion direction (Figure 2b) and are easily deformed by a GBS. Unlike coaxial grains, elongated grains form a mechanical pair that tend to rotate due to the stress direction. Hence the mechanism of GBS is encouraged [46]. GBS is a dominant induction process that results in inhomogeneous sliding in the microstructure, which is controlled by the diffusion mechanism [47]. Since a smooth boundary acts like a viscous liquid, the diffusion creep rates decrease with increasing boundary viscosity. During deformation, grain rotation occurs that is attributed to the GBS mechanism, due to shear stresses and elastic anisotropy of neighbor grains [47]. The slip effect on sliding can be substantial, while the intragranular slip is not a prior condition for GBS. Typically grains adjacent to one another deform dissimilarly and rarely similarly. To simplify the microscopic study, it can be supposed that each grain can be undergone only one slip system. The GBD generation is due to the dislocation dissociation of those which moved to grain boundaries from an upper grain because of the dislocation source absence in grain boundaries. Glissile GBDs can only move in a single direction and leave the crystal under shear stresses. Consequently, although sliding reaches an extent before the end of the boundary, it is inactive in the other one. For this reason, intragranular deformation dominates all sliding. The sliding occurs in both boundary ends by the generation of GBDs via grain boundary sources and it is increased from one boundary to the other. For grain identical deformation when boundary dislocation sources are inactive, no macroscopic sliding occurs. However, the movement of lattice dislocations to the boundaries induces opposite-sign glissile GBDs. These dislocations only induce local grain boundaries and move in opposite directions. When boundary sources generate grain boundaries, macroscopic sliding takes place. The generated stresses at boundaries can be relaxed by lattice dislocation migration into grains. As a result, the plain sliding rate is much lower than that of the grain boundary sliding [48]. At high stresses, the n was calculated to be 4.68 and 4.53 for the alloy and composite, respectively, which indicates a dislocation creep. In fact, with increasing stress, conditions have been provided for the movement of dislocations in prismatic and pyramidal planes in addition to the basal planes. On the other hand, at high temperatures, the diffusion rate is increased, allowing the dislocation climb.

**Figure 6:**
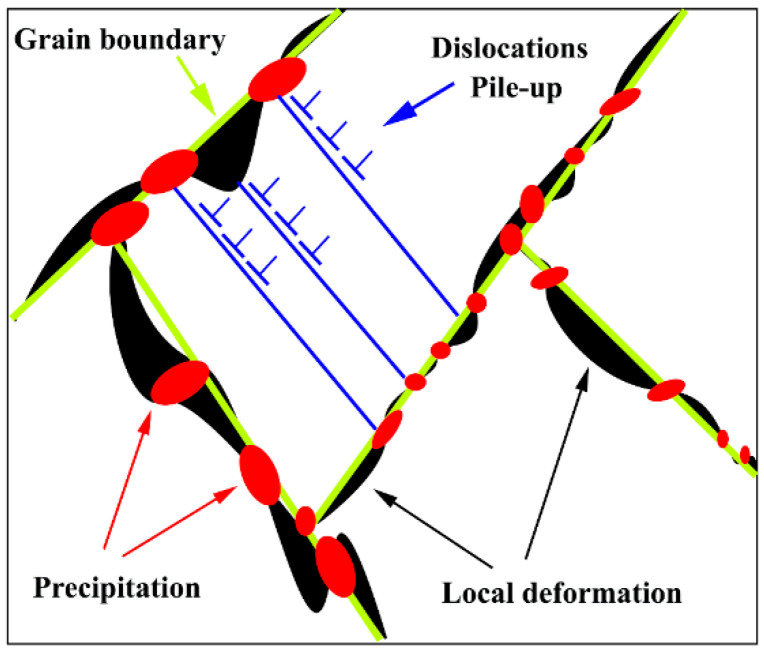
Schematic of precipitation strengthening of the grain boundary.

A comparison of the creep test results for the unreinforced alloy with pure Mg [49] shows that the creep strength and ductility of the ZK60 alloy are greater than that of pure Mg. In addition, when creep deformation is caused by GBS, a large deformation occurs in the specimen in which the specimens exhibit good ductility. In addition, the increase in creep strength is related to the presence of Zn in the alloy composition. The addition of Zn to the alloy increases the dislocation density by up to two-fold [36,43,50]. Moreover, the staking fault energy in the Mg alloy is reduced by the addition of the Zn, which results from the decomposition of the dislocation into partial dislocations and causes many plane defects in many (0001) planes. Zn segregation at the partial dislocations increases the partial dislocations distance. Therefore, the presence of the Zn element reduces the staking fault energy of the alloy, which makes the dislocation movement (especially the screw dislocation) more difficult. In addition, the presence of a Zr element with a larger atomic radius and a low solubility limit in Mg (at ambient temperature) results in solid solution strengthening, which is effective in improving the high temperature mechanical properties of the alloy [51]. The increase in strength can also be related to the presence of precipitation with thermally stable in the grain boundary (Figure 3). In fact, with the creation of stable precipitation at high temperatures around the grain boundary, creep resistance has improved, schematically illustrating the strengthening method in Figure 6. Given that the grain boundaries of Mg alloys are weak regions at high temperatures that can easily slide. On the other hand, the dislocations can easily cross the grain boundary into the neighbor grain. As a result, deformation occurs at high speeds in these areas and virtually causes high creep rates at high temperatures. According to Figure 6, the presence of precipitation in the grain boundary causes the grain boundary pinning, which makes the conditions difficult for GBS. On the other hand, the presence of precipitation in the grain boundary causes local deformation of the grain boundary and prevents dislocation movement that reduced the creep rate at high temperatures.

**Figure 7:**
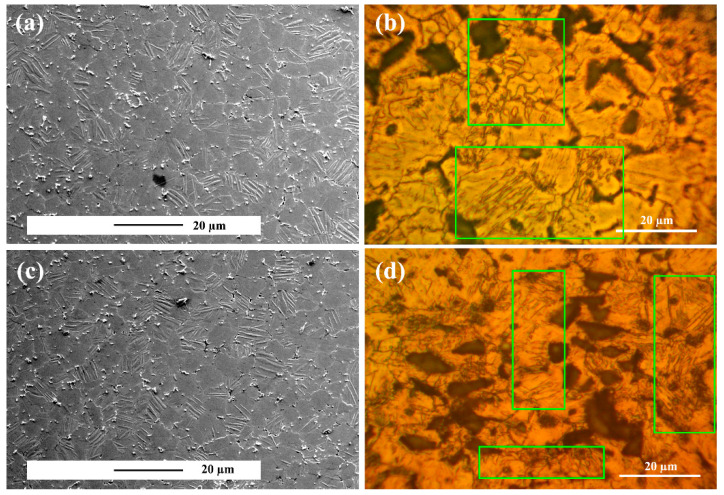
SEM micrographs of the microstructure after 25 h of creep test at 150 °C: (**a**) unreinforced alloy under 30 MPa stress, (**b**) ZK60/SiCp composite under 30 MPa stress, (**c**) unreinforced alloy under 60 MPa stress, and (**d**) ZK60/SiCp composite under 60 MPa stress.

By examining the alloy elongation after the creep test, an elongation of more than 18% at 30 and 60 MPa and increase to 23% at 80 MPa. Changes in the percent elongation of the composite also followed a similar trend to that of the unreinforced alloy. At all stresses, the elongation of the composite was about 30% lower than alloy due to the presence of particles in the microstructure. At low stresses (30 MPa) in the early stages of twinning deformation (Figure 7), the resulting increases elongation with increasing refinement. At moderate stresses (60 MPa) due to the double twinning, the resulting refinement increased in comparison to low stresses, which resulted in an increase in the percentage of elongation. At high stresses (80 MPa) the early stages of twinning deformation were not formed, and no twin-induced refinement occurred and the lack of grain size reduction and a noticeable increase in stress exponent have altered the deformation mechanism of the GBS to slip, which has reduced the ductility of the specimens. However, studies [52] have shown that the highest percentage of Mg elongation is obtained by activating the GBS mechanism. By comparing the sample elongation after the creep test at 150 °C with the superplastic behavior studies on ZK60 alloy, it is clear that the creep has a relatively low elongation, that can be related to the creep test temperature and microstructure heterogeneity. Moreover, the 150 °C was not enough for the samples to exhibit superplastic behavior. Microstructural changes, e.g., precipitate formation and recrystallization of the microstructure, lead to necking in the specimen, which prevents sensible elongation in the specimens.

### 3.3. Microstructure after Creep Test

Figure 7a shows the microstructure of the alloy crept under 30 MPa at 150 °C after 50 h. By comparing the microstructure of the crept sample compared to the initial microstructure of the samples, it is clear that the deformation in the sample is due to twinning. Due to the crystalline structure of magnesium (HCP), the plastic deformation in the two directions a and c is anisotropic, causing the activation energy of the slip to vary in different systems. This anisotropy is one of the causes of low temperature twinning [53,54,55,56,57]. Precipitates created during the aging process also affect twinning. In fact, the presence of precipitates in the microstructure increases twinning nucleation. In contrast, their size, growth rate, and volume fraction decrease, which increases the yield stress of the alloy [58,59]. Overall, with the twinning in the system, the grain size decreased, which based on the Hall–Petch relation increases the strength [60,61]. Figure 7b illustrates the composite microstructure crept under 30 MPa at 150 °C after 50 h. It is observed similarly in the composite that the deformation mechanism is twinning; however, the number of twins in the composite microstructure is lower than that of the alloy sample. The twin volume fraction in the composite can be related to the presence of particles in the structure. The presence of particles in the matrix causes effective load transfer, resulting in less uniform deformation in the composite matrix than in the unreinforced alloy. In addition, the presence of particles has led to refinement. Based on the modified Hall–Petch relation, twinning in small grains needs more stress [60,61]. The composite strengthening under low stresses is attributed to twins and grain refinement. Figure 7c and d illustrates the alloy and the composite microstructures crept under 60 MPa after 50 h. It is seen in the figure at 60 MPa stress is also the deformation mechanism for unreinforced alloy and composite twinning. By comparing Figure 7a–c, it is clear that by increasing stress, the twinning content has increased. Furthermore, we observed in the unreinforced alloy microstructure that secondary twinning was created. In fact, with increasing stress, the energy needed to nucleate secondary twins on primary twins is provided.

**Figure 8:**
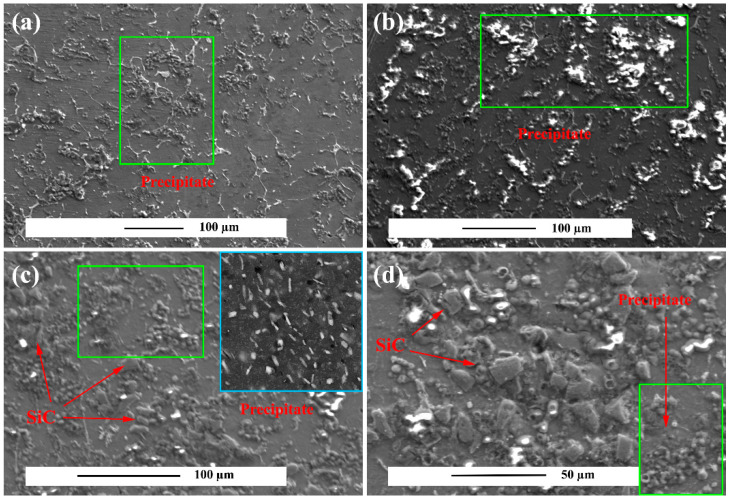
Scanning electron microscopic image of the microstructure after creep test at 150 °C under 100 MPa stress: (**a**) unreinforced alloy under 30 MPa, (**b**) unreinforced alloy under 100 MPa, (**c**) ZK60/SiCp composite under 100 MPa, and (**d**) ZK60/SiCp composite under 100 MPa.

**Figure 9:**
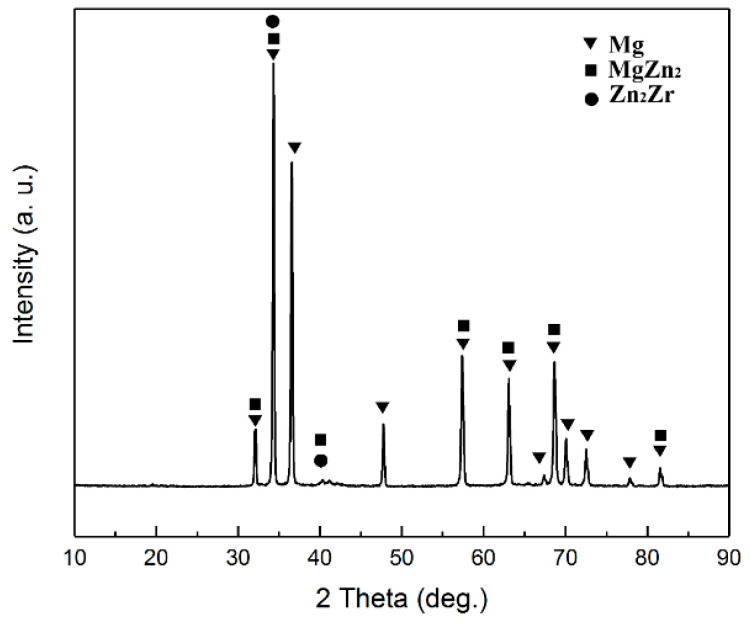
X-ray diffraction pattern before and after 50 h of creep at 150 °C under 100 MPa stress.

Figure 8 shows the alloy and composite microstructure alloy crept under 30 and 100 MPa after 50 h. Figure 8b,e show that precipitates in the microstructure with uniform distribution were created during the creep test, indicating the activation of the dynamic precipitation process. Precipitates created inside the grain have two different morphologies. One is disk-like precipitates of 50–100 nm size and the other is rod-like precipitates of 100–200 nm size as shown in higher magnification figure. These precipitates are formed due to the local saturation of the dissolved atoms during creep. To determine the type of precipitates, the sample was subjected to XRD, as shown in Figure 9, indicating the presence of two $\beta_{1}^{\prime }\left({\mathrm{MgZn}}_{2}\right)$ and $\beta_{2}^{\prime }\left({\mathrm{Zn}}_{2}\mathrm{Zr}\right)$ precipitates in the microstructure. According to the conducted study [62], it is clear that the melting point of $\beta_{1}^{\prime }\left({\mathrm{MgZn}}_{2}\right)$ and $\beta_{2}^{\prime }\left({\mathrm{Zn}}_{2}\mathrm{Zr}\right)$ precipitates is equal to 330 and 465 °C, respectively, which indicates the stability of the precipitates in the creep test temperature and the strengthening effect of the precipitates. According to thermodynamic and kinetic calculations on the precipitates’ formation in the ZK60 alloy, because Zr has a higher affinity than Mg, the $\beta_{2}^{\prime }\left({\mathrm{Zn}}_{2}\mathrm{Zr}\right)$ precipitates first, and by reducing the Zr concentration around it, the residual Zn in the alloy reacts with Mg and gives rise to $\beta_{1}^{\prime }\left({\mathrm{MgZn}}_{2}\right)$. The shape of the precipitates $\beta_{1}^{\prime }\left({\mathrm{MgZn}}_{2}\right)$ is a rod that grows in planes (-10–12) and in the direction [0001] (c direction) of the Mg crystal structure and has a constant length-to-diameter ratio [19]. The shape of the precipitates $\beta_{2}^{\prime }\left({\mathrm{Zn}}_{2}\mathrm{Zr}\right)$ disk growing on the planes (0001). In fact, temperature and stress creep tests have provided the conditions for dynamic precipitation. According to the calculated stress exponent at 150 °C, it is evident that the slope of the graph has changed at high stresses. In fact, the creep rate change is due to the interaction of dislocations with precipitation, which causes threshold stresses during creep. Studies [19] have shown that dislocations/rod precipitates interaction is more probably. By comparing the images of the unreinforced alloy and composite in Figure 8 it is apparent that the density of the precipitates is different in the unreinforced and composite alloy. Based on the calculations made by XRD, it was determined that the percentage of precipitates in the unreinforced alloy is 15% and in the composite is 25%, which justifies the different creep rates created for the unreinforced alloy and composite.

**Figure 10:**
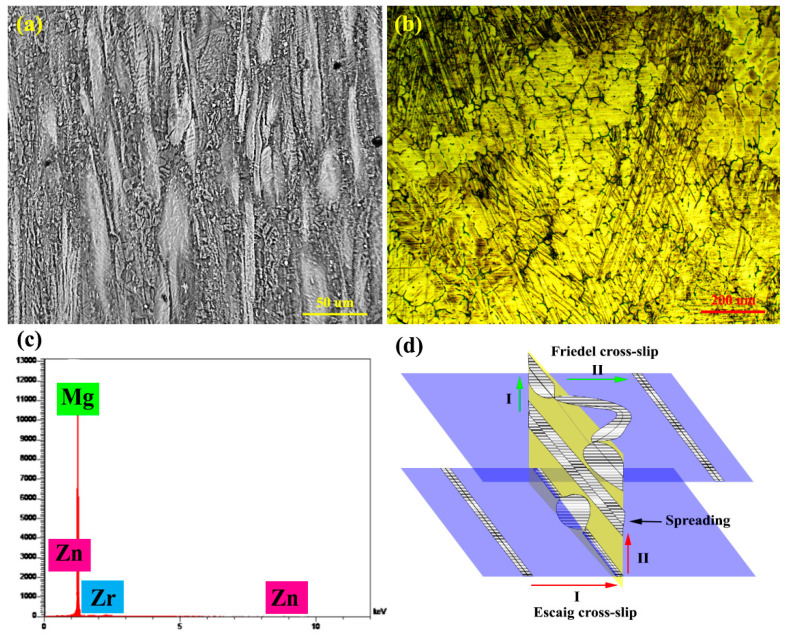
(**a**,**c**) SEM micrograph and EDS analysis, (**b**) OM micrograph after creep at 150 °C under 100 MPa stress, and (**d**) schematic of the DFE mechanism.

Supersaturated zones are created for dynamic deposition by moving dislocations at high stresses. In fact, at high stresses dislocations are released from the atmosphere of dissolved atoms and create supersaturated zones. These supersaturated zones are due to the dynamic aging process (DSA). On the other hand, activation of the DSA mechanism due to frictional stress due to dislocation motion in the grain increases the strength of the sample. The high applied stress to the sample provides the activation energy by the formation of required vacancies and rapid diffusion of solute atoms via the DSA mechanism. The DSA activation energy depends on the diameter of the soluble atoms, that is stated to be 63–108 kJ/mol [63], which is in agreement with the activation energy under high stresses for alloy and composite (QZK60 = 135.58 and QZK60/SiC = 112.38 kJ/mol). By comparing the microstructure at 60 and 100 MPa stresses, twins are not formed in the structure as the stress increases, because dynamic precipitation activation and the resulting mismatch resulting from precipitates provide the conditions for the absorption or creation of dislocations and the deformation caused by the dislocation motion prevents the stress concentration from forming twinning in the microstructure [64]. Furthermore, by comparing the initial microstructure of the sample with the unreinforced alloy microstructure crept under 100 MPa stress, slip lines are visible (Figure 10), that can be due to the double Friedel–Escaig (DFE) cross slip mechanism. The DFE mechanism occurs in basal and/or non-basal planes. Based on Figure 10d the screw dislocation to change the slip plane in two stages, first entered into the plane perpendicular to the two planes, and when the dislocation is locked in the plane perpendicular to the primary plane and it spreads, which is called the Escaig stage. Screw dislocation breaks down from the plane on which it is located to the parallel plane of the first plane, and creates a dislocation loop on a plane perpendicular to the two parallel planes. The driving force behind the dislocation loop is the external stress that is exerted on the specimen. The dislocation decomposition causes two edges in the plane perpendicular to the first plane to cause the dislocation wave motion, in which at this temperature there is the activation energy for the dislocation wave motion. Then it is unlocked in two stages. The displacement wave causes the lines in the microstructure [65], which can be seen in Figure 10a,b. Studies [66] have reported the occurrence of this mechanism at 2.4 × 10^−3^ normal stress accommodated with Q = 105 kJ/mol, that is near the Q value of the alloy and composite samples.

### 3.4. Texture

**Figure 11:**
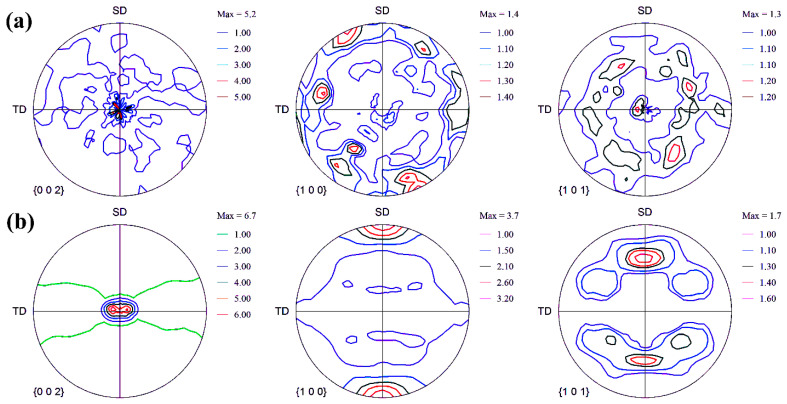
Pole figures of SD samples crept at 150 °C: (**a**) unreinforced alloy, and (**b**) composite sample.

In order to study the deformation behavior of Mg alloys and composites in more detail, the texture of samples that have undergone the creep process has been examined. For this purpose, polar images of basal planes {0002} and non-basal planes {10–10} and {10–11} of the magnesium alloy and composite specimens subjected to the creep process are shown in Figure 11. It should be noted that in all the polar planes shown in Figure 11, the SD is the extrusion direction, TD indicates the transverse direction, and ND is the normal direction, which is located in the center of the polar image. As can be seen in the polar image of the basal planes (0002), the densities of many poles are at the center of the polar image. This texture indicates that the basal planes of many grains are perpendicular to the normal direction. In other words, the grains are oriented so that their c-axis is parallel to the normal direction. Such a texture has been reported by Zhang et al. [67] to investigate the effect of primary texture on the anisotropic creep behavior of Mg-Y alloy sheets. A comparison of polar images of the unreinforced sample (Figure 11a) and the composite sample (Figure 11b) shows that the texture intensity of the basal planes of the composite sample is higher than that of the unreinforced alloy. The maximum texture intensity of the basal planes of the unreinforced alloy is 2.5 and that of the composite sample is 6.7. The higher intensity of the basal texture of the composite specimen than the alloy specimen indicates that the larger volume fractions of the grains of the composite specimen are oriented so that their basal planes are parallel to the SD. The reason for the difference in texture intensity in magnesium samples may be related to the different deformation mechanisms that were activated during the creep test [68].

In the case of polycrystalline magnesium alloys, the deformation of the material is different from that of cubic metals due to the constraints imposed by the grains adjacent to the grain boundaries. Activation of the slip system only leads to the activation of two different slip systems. However, the homogeneous deformation of polycrystalline structures requires five independent slip systems, which is not possible only by activating the slip systems of the basal and the prismatic, which lead to the adjustment of the strain in the <a> direction. According to Taylor theory, for uniform plastic deformation, when the dislocations reach the grain boundaries, slip from one grain to the adjacent grain is not possible due to the misalignment of the slip planes. Therefore, at least five independent slip systems are required to prevent failure at the grain boundaries. Regarding magnesium slip systems, it can be said that there are three slip systems in the basal plans <a>, {0001} <11–20>, of which only two systems can operate independently of each other [69]. Since this combination of the slip plane and direction is similar to other systems. Similarly, the two prismatic slip systems <a>, {1–100} <11–20>, operate independently of the three prismatic slip systems <a> [69]. Therefore, in order to create strain in the direction of the c-axis, magnesium alloys require additional deformation, i.e., activation of the pyramidal slip system or twins, which causes the strain to align in the <c + a> direction. Therefore, it requires additional deformation, i.e., activation of the pyramid slip system or twins, which causes the strain to adjust in the <c + a> direction. In other words, because each grain cannot provide five independent slip systems, adjacent grains with different orientations cannot completely deform the material, leading to strain matching at the grain boundaries. Therefore, dislocations pile-up at the grain boundaries because they are unable to cross the grain boundaries. This leads to localization of stress at the site of pile-up dislocations, which eventually activates non-basal slip systems, twin or failure [70,71].

It should be noted that the critical resolved shear stress (CRSS) or the minimum stress required for material flow due to the activation of the deformation mechanism for basal plane slip is very low and about 0.5–0.7 MPa, while CRSS for the system non-basal slip at low temperatures (<200 °C) is higher than 40 MPa. As can be seen, this value is much higher for activating non-basal slip systems than for non-basal slip systems, and therefore the predominant deformation mechanism under such conditions is non-basal slip systems. However, the CRSS value for non-basal slip systems at higher temperatures (>200 °C) is 2–3 MPa due to the thermal dependence of dislocations on the non-basal slip [72,73]. According to the microstructure of the alloy shown in Figure 7, it can be seen that twins are formed inside many grains. In general, three types of twins have been reported in magnesium alloys: tensile twins, compressive twins, and double twins. Tensile twin leads to tensile strains along the c-axis and is activated when a tensile strain is applied parallel to the c-axis or pressure perpendicular to the c-axis. CRSS is the lowest value (2–3 MPa) for basal slip, and these twins can be activated in the early stages of deformity. Compressive twins, {10–11} and {10–13} are activated when a compressive strain is parallel to the c-axis or a tensile strain perpendicular to the c-axis is applied. Tensile twins {10–12} and compressive twins {10–11} cause rotation of the basal plane to 86° and 56°, respectively, to a state in which there are no twins. When in the early stages of deformation, first compression twins {10–11} or {10–13} is formed first and then a tensile twin {10–12} is formed through the twinned area, a double twin {10–11}{10–12} or {10–13}{10–12} is formed. Double twin causes pressure along the c-axis and rotation of the basal planes by 38° [74,75].

**Figure 12:**
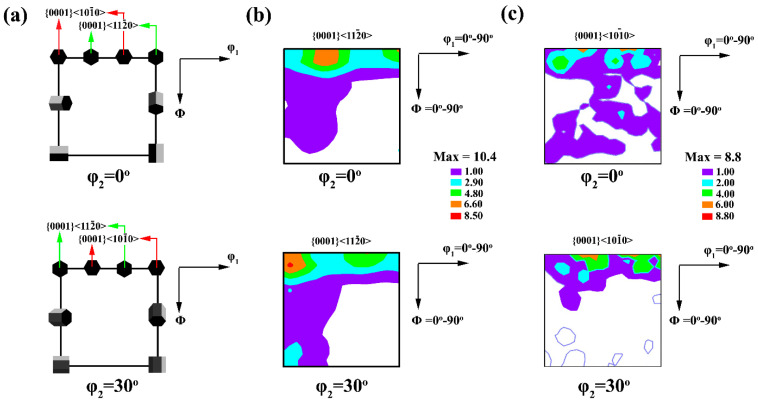
ODFs of the crept samples at ϕ_2_ = 0° and 30° sections: (**a**) schematic of the ideal texture components, (**b**) unreinforced alloy, and (**c**) composite sample.

According to the explanations given and the study of the strong texture of the basal plane and the weak texture of the non-basal plane in both the alloy sample and the composite sample that underwent the creep process, it can be seen that there are areas with good orientation for twin formation. Since most of the poles are in the center of the image, it is expected that the sample texture before the creep process is weak (random texture) so that the poles are randomly located across the polar image. In other words, the sample texture before the creep process was such that the c-axis of the grains was perpendicular to ND. After the creep process, due to the presence of multiple twins in the microstructure (Figure 7) and to apply load during the creep process and the shape of the twins, which are lens-shaped and thick, the formed extension twins are expected. As mentioned earlier, the activation of the extension twins causes the basal plane to rotate 86° relative to the original position. Under such conditions, the grains are oriented in such a way that the c-axis of the grains is parallel to the ND or perpendicular to the SD or extruded direction. In this case, the grains are oriented in such a way that the base planes are parallel to the SD or perpendicular to the ND. The observation of the weak texture of the non-basal planes also confirms the activity of the basal plane and the formation of twins, which were activated in order to facilitate the deformation process in such conditions. A comparison of the texture strength of the basal plane of alloy and composite samples indicates that the intensity of the basal texture of the alloy sample is lower than that of the composite sample. The reason for this may be related to the volume fraction of the formed twins and the primary texture. The decrease in texture intensity due to twins is related to the rotation of the lattice planes by the tensile twins, so the lower the volume fraction of the twins, the lower the texture intensity due to the twins. However, the fact that the movement of dislocations between the basal plates generally leads to the rotation of the crystal in the direction that the c-axis of the grains is parallel to the extrusion direction [74], leads to strengthening the basal texture. Therefore, it can be expected that the activity of the basal slip system in the composite sample is higher than in the alloy sample. Furthermore, dislocations climb between adjacent basal planes can also increase the intensity and concentration of the basal texture in the center of the polar image [74]. Examination of the texture of (10–11) plans of the alloy and composite samples in Figure 11 shows that the texture of non-basal planes of composite samples is more than that of alloy samples. This may be due to the fact that in HCP structures, prismatic slip <a> has a component similar to the basal slip <a>, and when the prismatic component <a> is combined with the basal component <a>, they lead to cross-slip between the base and prismatic planes. Thus, cross-slip can occur in {10–11}<2110> that the Burger vector is the sum of the Burger vector of the basal and pyramidal slip systems [76,77]. Figure 12 illustrates the orientation distribution functions (ODFs) of the samples after the creep test for φ_2_ = 0° and 30° sections. Furthermore, the ideal position of the texture components is shown schematically in Figure 12. The unreinforced alloy showed a moderately strong fiber texture along the angle of ϕ_1_ = 0–90°, which was tilted about Φ = 10°. This implied the tilting of the basal poles from the ND by 10°. The major components of the texture were rotated in the angle range of ϕ_1_ ≈ 5–10° from the standard positions of both texture components of (0001)<11–20> and (0001)<10–10>. It confirmed that the texture of the samples, which had undergone symmetric crept could present both (0001)<11–20> and (0001)<10–10> crystallographic orientations. However, the main texture components proposed that the texture was dominated by the TD spreading. This is consistent with the basal pole figure of (0002) in Figure 11.

The texture of (0001)<11–20> for the composite sample, which was crept initiated the domination of the major components, which was shown at (30°, 10°, 0°) and (0°, 10°, 30°). However, the basal poles were tilted by ~10° towards TD. However, a new strong texture component was observed at (90°, 5°, 0°) for the composite sample, which crept due to minor splitting of the basal pole by ~5° toward RD. According to Figure 11, although, the composite displayed the basal texture after creeping at 150 °C, the intensity was significantly enhanced compared to the unreinforced alloy. The basal texture spread to RD or TD was observed for unreinforced samples. Regarding the ODF sections in Figure 12, the crept sample only displayed the basal pole tilting. During the RD splitting of basal poles, the strongest texture components were obtained at (90°, 10°, 30°) and (90°, 10°, 0°). While the main fraction of (0002) poles of the crept sample was aligned along the ND. Additionally, the texture of the samples had a combination of two crystallographic orientations of (0001)<11–20> and (0001)<10–10>. This was revealed by the strong texture components at (60°, 0°, 30°), (30°, 0°, 0°), (0°, 0°, 30°), and (90°, 0°, 0°) as characteristics of (0001)<11–20> texture.

### 3.5. Damage Mechanism

#### 3.5.1. Perpendicular to the Fracture Surface

**Figure 13:**
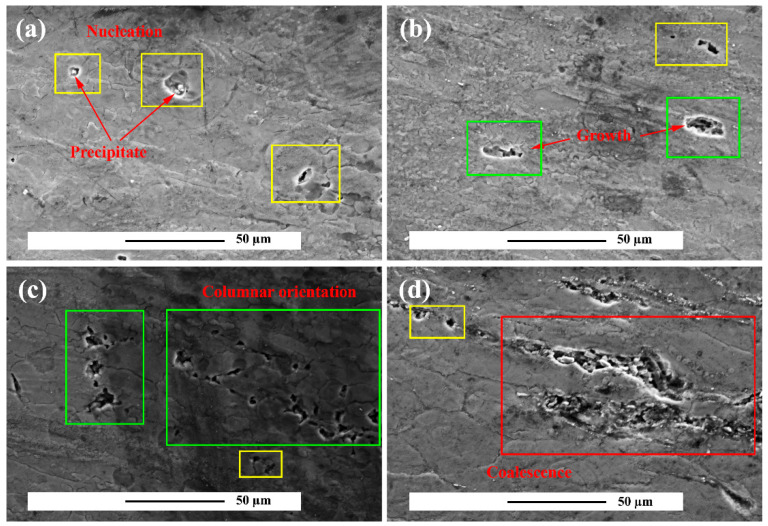
Cavitation stage in unreinforced alloy after creep test at 150 °C under 60 MPa stress for times of: (**a**) 72 h, (**b**) 96 h, (**c**) 120 h, and (**d**) 144 h.

The microstructure changes in the unreinforced alloy in the creep test under 30 MPa stress at 150 °C at different times are shown in Figure 13. According to Figure 13a, the first stage of defect initiation is the nucleation of the voids. At the second stage of creep, the cavities began to nucleate and over time the cavities grew on one side and the nucleation speed on the other increased (Figure 13b). Further deformation of the cavities occurred and their alignment was perpendicular to the loading direction (Figure 13c) and the cavitation coalescence resulted in cracking (Figure 13d). According to Figure 13a, macroscopic fracture crack growth occurred. Based on these observations, the degradation process for the unreinforced alloy can be classified into III stages. Latency stage, nucleation, and growth defect.

**Figure 14:**
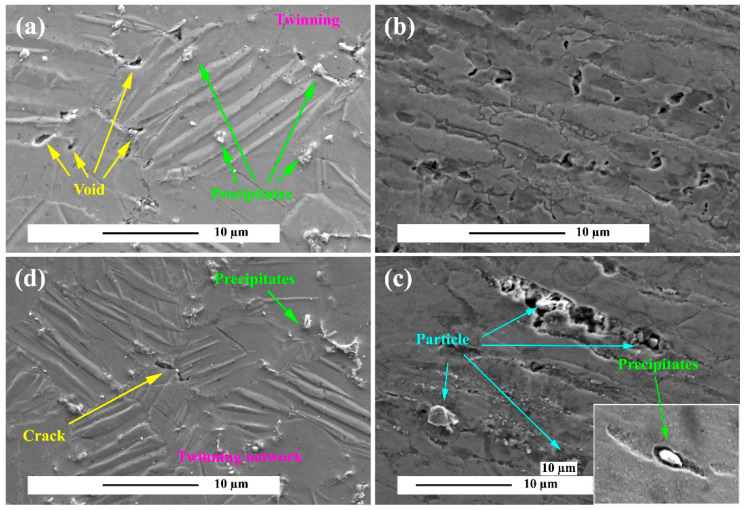
Formation of cavities and cracks at the twin and grain boundaries after creep at 150 °C under 39 MPa stress in the sample: (**a**,**b**) unreinforced alloy, (**c**,**d**) ZK60/SiCp composite.

The microstructure of the areas around the fracture surface of the alloy and composite is illustrated crept at 150 °C under 30 MPa in Figure 14. Figure 14a illustrates the twinning aggregates in the microstructure, which is at the interface between the precipitates where the void nucleation at the twinning intersection and the twinning and precipitates site intersection. Moreover shown in Figure 14b are the prior particle boundary cracks (PPB) caused by the pile-ups of dislocations at this temperature. The PPB by crating the vortex structure that causes the void. In the composite microstructure in Figure 14c, it is clear that there is an intense interaction between the mechanical twinning and the grain boundaries at the microscopic scale until twinning saturation. It is clear from the figure that the twinning edges are a prone location for stress concentration and voids nucleation. Twinning saturation in the microstructure has led to the growth of voids in the stressful grain boundaries and ternary grain boundaries. It is also evident in the figure that the cavities were formed at the intersection of the precipitates and twinning. In fact, the difference between elastic modulus and thermal incompatibility between matrix/particle and matrix/precipitation causes the localization of the flowing and voids formation in the composite. A series of cavities formed in various microstructural components rapidly intertwine and produce macroscopic cracks that lead to necking [78,79,80,81]. According to Figure 14c, it is evident that wedge cracks in the composite microstructure also occur. During continuous deformation at high temperatures, the amount of heat produced is very high, so the composite does not have the time to adapt to the high tensile stress created near the interface, which results in nucleation, coalescence, and formation of wedge cracks in the microstructure. Also shown in Figure 14d is a grain boundary crack. The highest concentration of plastic strain in Mg alloys occurs in so-called hard grains where the planes {11–20} and {10–10} are perpendicular to the stress direction and are preferred for twin deformation. The extreme localization of plastic deformation in the grains results in a high stress concentration in the grain boundary. This leads to the nucleation and/or cavities coalescence that causes the growth of intergranular cracks. Although cracks in precipitates begin at an early stage, their growth during deformation is limited [82,83]. The crack growth stage begins by coalescence of the cracks in the microstructure, leading to the onset of macroscopic failure [84,85].

#### 3.5.2. Fracture Surface

**Figure 15:**
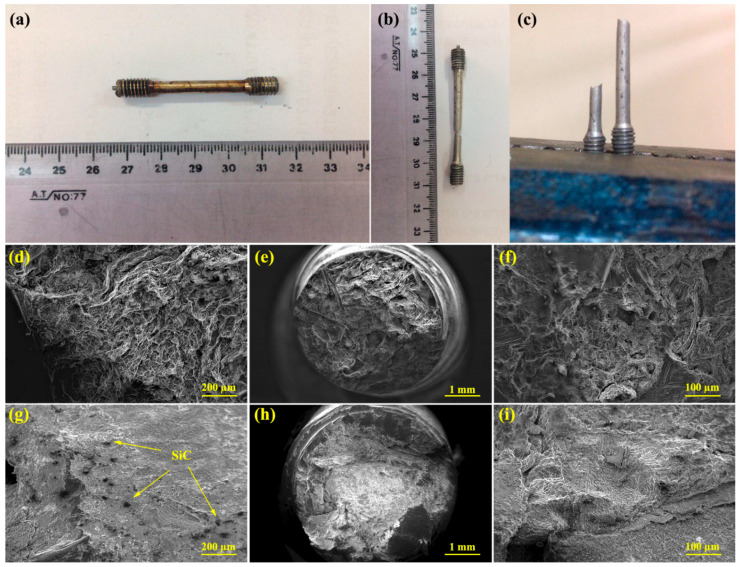
(**a**) Sample before creep test, (**b**,**c**) fracture sample after creep test, (**d**–**f**) fracture surfaces of the unreinforced alloy after creep test at 150 °C under 30 MPa stress, and (**g**–**i**) fracture surfaces of the ZK60/SiC_p_ composite after creep test at 150 °C under 30 MPa stress.

Figure 15a–c show the sample before and after creep test. Figure 15b illustrates of the sample showing the location of the fracture area in the gage length. A photograph of the cross section received for analysis is shown in Figure 15c. Figure 15d–f show SEM images of the fracture surface of the alloy crept under 30 MPa. Examination of fracture surfaces shows a cup-like feature with high fraction of microvoids that are of different sizes and are characterized by ductile fracture. The accumulation of microvoids tends to ductile fracture. At the ductile fracture surface, stretched dimples can be seen in the stress direction, indicating high plastic deformation. According to the figure, the shear lip zone and the fiber zone are observed at the outer periphery and the central region of the fracture surface, respectively. Moreover, flat surfaces are evident on the shear-lop zone of the outer periphery, consisting of shear dimples caused by the shear stress. On the other hand, the appeared equiaxed fine and deep dimples approve that the creep exposure results in dimple rupture (ductile fracture). Furthermore, in the fiber zone, equiaxed dimples are evident, confirming the occurrence of ductile fracture. Based on the fracture surface, creep deformation of the unreinforced alloy was dominated by the ductile fracture mechanism. While the fracture surface of the composite sample displayed the shear-lip zone. Nevertheless, the shear-lip zone was absent for unreinforced alloy. The presence of the fiber zone at the fracture surface after all creep tests implies the occurrence of transgranular fracture. However, the fine and deep dimples density and their sizes were different for the composite and unreinforced alloy samples. The composite sample showed more fine dimples at high stress levels. Large plastic deformations were found to be associated with the fiber zone slip lines. As per the high-magnification micrograph (Figure 15i) obtained from the fiber zone, both deep and fine dimples can be seen at the fracture surface. 

Figure 15g–i show an SEM image of the composite fracture surface at 30 MPa. At the composite fracture surface, such as unreinforced alloy, the presence of dimples is evident due to cavitation during creep. The image also shows brittle fracture areas due to the presence of particles and a tendency to brittle fracture. In Figure 15i, the presence of precipitation is evident at high magnification, indicating the cavities’ formation and crack growth in the precipitates/matrix interface. During deformation at high temperatures, the microvoid does not rapidly coalescence together, but instead forms micro-necking, forming filaments that crack when the filaments break. These filaments are bridges between the microvoids. There is no apparent debonding at the composite fracture surface, indicating a strong particle/matrix interface. However, there is no debonding around the fracture surface. The crack growth started from part of the specimen and after localized specimen failure, loads were applied to the particles around the surface causing energy to be provided for the debonding of the particle. In Figure 15g, a high magnification debonding of a particle at the fracture surface is illustrated, which is observed around the particle of brittle fracture zones containing microcracks. The existence of these microcracks can be due to the stress concentration at the particle edge. The creation of fine-grained pile-up of the dislocations on the plane near the Lamela boundary. In fact, the matrix/particle elastic modulus difference causes a pile-up the dislocations in the hard phase/soft phase interface, where the high density of the dislocations causes the localization of deformation and with increasing stress concentration, cracks grow [86,87,88,89]. Moreover, precipitates can be present adjacent to deep dimples. Typically, reinforcement particles and precipitates are harder than the matrix, resulting in presence of a deformation gradient between the matrix and the reinforcement or precipitates under creep conditions. Therefore, precipitates can hasten the fracture by developing crack-nucleation-preferred sites. The precipitates are mostly close to lath/grain boundaries, while lower-stress fractures may be due to brittle intergranular failures. In addition, stress concentration around coarse precipitates/reinforcement SiC particles. The elevated localized stress at the particle/matrix interface results in extreme plastic deformation, causing micro-crack formation [90,91].

## 4. Conclusions

The creep behaviors of unreinforced ZK60 alloy and the composite of ZK60/SiC_p_ were investigated after the KoBo extrusion and aging process at 150 °C and various stress levels. By examining the microstructures and analyzing the creep data, the dominated creep mechanism and the mechanism of damage are introduced. The results can be summarized as follows:

1. Bimodal grain distribution is achieved in the alloy and composite microstructures during KoBo extrusion, in which the grain sizes were less than 5 µm.
2. The results of the creep tests showed that parameters n and Q_c_ of the samples were altered at high-stress conditions, showing the change of GBS mechanism to dislocation climb.
3. It was found that pre-aging at 175 °C can achieve double aging at 150 °C under high stresses which caused precipitates growth.
4. The strengthening mechanism has been the twinning, double twinning, and dynamic precipitation at low, moderate, and high stresses, respectively.
5. The failure mechanism was cavitation at the tertiary grain boundaries and at the intersection of the twinning with the grain boundary and the precipitates.
6. Evaluation of the elongation of the samples showed that due to the low temperature and microstructural heterogeneity, premature necking occurred and caused the elongation percentage not to exceed 23%.
7. The unreinforced alloy showed a moderately strong fiber texture along the angle of ϕ_1_ = 0–90°, which was tilted about Φ = 10°. A new strong texture component was observed at (90°, 5°, 0°) for the composite sample, which crept due to minor splitting of the basal pole by ~5° toward RD.

## Figures and Tables

**Table 1:** Mechanical properties and grain size of the KoBo extruded and aged samples.

	UTS (MPa)	YS (MPa)	Elongation (%)	Grain Size (µm)
ZK60 alloy	265	230	15	10
ZK60/SiC_p_ composite	365	320	6	5

## Data Availability

Not applicable.
